# Investigation of the Changes in Microstructure and Transport Properties of Leached Clay–Cement Pastes

**DOI:** 10.3390/ma19142937

**Published:** 2026-07-08

**Authors:** Kailai Zhang, Wenwei Li, Huamei Yang, Xinyu Li, Dan Tian, Fan Li

**Affiliations:** Hubei Key Laboratory of Water Engineering Materials and Application Technology, China Three Gorges Corporation, No. 1, Liuhe Road, Wuhan 430014, China

**Keywords:** leaching, clay–cement paste, microstructure, diffusion coefficient, permeability coefficient

## Abstract

Clay–cement slurry, as a widely used anti-seepage material, is prone to calcium leaching and deterioration when exposed to environmental water. The influence of microstructural and mineralogical evolution on the transport properties of clay–cement samples under leaching conditions remains to be investigated. In this paper, accelerated calcium leaching tests were conducted on clay–cement pastes. A variety of techniques, including XRD, SEM, and NMR, were used to characterize the microstructural and mineralogical changes in the leached samples. The effect of accelerated leaching on transport behavior was studied by measuring changes in the water permeability and calculating diffusivity. XRD and SEM analyses show that after 28 days, the characteristic peaks of portlandite and ettringite almost disappear, while C-S-H gel undergoes decalcification and decomposition, leading to an increase in pore number and a notable rise in pore size (up to 1.90 μm). NMR results indicate that total porosity and peak pore size increase significantly, with the proportion of gel pores decreasing and that of small capillary pores (10–50 nm) rising from 10% to 22.1%. Moreover, the surface layer porosity (0–5 mm) increases from 31.33% to 50.65%, while the middle and lower layers show less degradation, indicating a progressive deterioration pattern. Regarding transport properties, the hydraulic conductivity increases from 4.7 × 10^−10^ cm/s to 2.14 × 10^−8^ cm/s (a two-order-of-magnitude increase), and the diffusion coefficient rises from 1.6 × 10^−11^ m^2^/s to 8.6 × 10^−11^ m^2^/s (a 5.3-fold increase). Both the diffusion coefficient and its increase factor gradually decrease from the surface to the interior, consistent with the evolution of porosity.

## 1. Introduction

Clay–cement slurry exhibits excellent stability and effective anti-seepage and plugging performance. It is non-polluting and reduces cement consumption. This material has been successfully applied in dam curtain grouting, reservoir anti-seepage reinforcement, and landfill grouting projects. Over their long terms of service, the decomposition of calcium compounds, such as calcium hydroxide (CH) and calcium silicate hydrate gel (C-S-H), is evitable since the pH of the aqueous environment (pH = 7) is much lower than that of the cement-based material’s pore solution (pH = 12.5–13 [1,2]). This phenomenon is referred to as calcium leaching. Calcium leaching leads to an increase in the porosity, permeability coefficient, and diffusion coefficient of the material, thereby reducing its mechanical properties and affecting engineering safety and performance.

Many long-operating dams have experienced leaching problems [3,4,5]. At the Guanyinyan Hydropower Station, five years after cement grouting was applied to the dam foundation of monolith No. 10 on the left bank, the filling ratio of cement stone in the grouting gallery decreased from 95.7% to 61.2%. On the left bank at an elevation of 985 m, the filling ratio of cement stone in the grouting gallery dropped from 100% to 70.6%. This indicates that calcium leaching caused erosive damage to the anti-seepage curtain, thereby reducing its durability [6].

Calcium leaching is a research hotspot in concrete durability, and many scholars worldwide have conducted related work [7,8,9,10,11,12,13,14]. Stephanie [15] conducted leaching degradation tests on cement paste specimens at different temperatures (26 °C, 72 °C, 85 °C) and established simplified formulas for leaching depth under various temperatures. LeBellego et al. [16] conducted a study on the effects of chemical–mechanical factors on the stiffness and strength of mortar beam specimens of varying sizes. Calcium leaching tests were performed in an ammonium nitrate solution at a concentration of 480 g/L. The results indicated that the stiffness, maximum load-bearing capacity, and fracture energy of the mortar beams significantly decreased during the leaching process. Heukamp [17] investigated the mechanical properties of cement slurry specimens after corrosion through triaxial tests. The results showed that the strength and internal friction of the specimens significantly decreased after corrosion, and they became more sensitive to changes in pore water pressure, exhibiting characteristics of soil. Phung [1] investigated the microstructural changes of cement paste specimens with different water-to-cement ratios and limestone powder additions during the leaching process. The results showed that leaching significantly altered the microstructure of the cement paste, transforming it into a material with a higher specific surface area, greater porosity, and a pore size distribution shifted toward larger pores. As mentioned above, research on the leaching of cement-based materials has primarily focused on the leaching depth and post-leaching mechanical properties. In contrast, studies on the evolution of microstructure during leaching and its correlation with transport properties remain relatively scarce.

In real service environments, calcium leaching is a gradual process and causes composition, structure and property gradients. For the characterization of concrete at the meso- and microstructural levels, a variety of techniques can be employed, such as scanning electron microscopy (SEM) [18,19], mercury intrusion porosimetry (MIP) [20,21,22], computed tomography (CT) [23,24], thermogravimetric analysis (TGA) [25,26], nuclear magnetic resonance (NMR) [27,28], and X-ray diffraction analysis (XRD) [29,30]. In laboratory settings, using water as the erosion medium makes it difficult to observe leaching degradation phenomena in the short term. Instead, continued hydration of cement can enhance material performance, which is entirely contrary to actual conditions [14]. Therefore, physical tests often employ accelerated leaching methods, such as using chemical reagents (ammonium chloride [31,32,33], ammonium nitrate [34,35,36]) or electro-accelerated testing methods [37,38,39,40]. Currently, the most widely used methods domestically and internationally involve applying 6 mol/L solutions of ammonium nitrate or ammonium chloride. Given that pore structure is closely associated with the macroscopic performance of materials [41,42], a systematic investigation of its evolution under accelerated leaching conditions is warranted.

The degree of leaching is closely related to the leaching depth, and specimens from different leaching zones exhibit remarkable differences in pore structure and transport properties (e.g., permeability and diffusivity). Nevertheless, most existing studies have treated leached specimens as homogeneous wholes when conducting post-leaching tests [43,44], with only a few investigations addressing microstructural variations at different leaching depths [2]. Meanwhile, clay–cement slurry, as a widely used anti-seepage material, is highly susceptible to deterioration due to environmental water leaching. To date, research on leaching behavior has predominantly focused on pure cement grout or systems incorporating mineral admixtures such as limestone powder [2] and fly ash [45], etc. Systematic studies on the leaching-induced degradation of clay–cement slurry remain unreported. The introduction of clay components further complicates the leaching process of cementitious materials, as the interaction between clay minerals and cement hydration products—and its influence on the leaching kinetics and microstructural evolution—requires further elucidation.

In this paper, accelerated calcium leaching tests were conducted on clay–cement pastes, and the evolution of microstructures at different locations was systematically investigated. The experimental procedure was as follows: first, the clay–cement pastes were subjected to leaching treatment in an aggressive environment (6 mol/L NH_4_Cl solution) followed by sectional sampling; subsequently, a series of quantitative and qualitative analyses were performed, including scanning electron microscopy (SEM), nuclear magnetic resonance (NMR), X-ray diffraction (XRD), and permeability coefficient tests; finally, the diffusion coefficients of the material at different locations were calculated and analyzed.

## 2. Materials and Methods

### 2.1. Materials

The cement-clay slurry prepared in this test used Hailuo P·O42.5 ordinary Portland cement (produced by Nanjing Conch Cement Co., Ltd., Nanjing, China). The clay was obtained from the core wall of an extra-high earth-rockfill dam (dam height: 295 m), and the mixing water was deionized water (pH = 7). The chemical composition and properties of the cement are presented in Table 1 and Table 2, respectively. The chemical composition of the clay is shown in Table 3. The mix proportion of the slurry used in the test is listed in Table 4.

### 2.2. Methods

According to the designed mix proportion, the cement-clay slurry was prepared, mixed, cast into molds, and then cured by immersion in a saturated calcium hydroxide solution. After the curing period, to investigate the one-dimensional calcium leaching behavior of the cement-clay slurry, the specimens were sealed with waterproof latex. The molding and curing of all specimens were carried out in a constant-temperature environment at 20 °C. The overall porosity and permeability coefficient of the specimens were measured at 7, 14, 21, and 28 days. After 28 days of leaching, the specimens were sectioned for sampling at depths of 0–5 mm, 10–15 mm, and 15–20 mm. For samples obtained from different depths, macro- and micro-scale tests were conducted, mainly including pore structure, surface morphology, and phase composition.

In this study, 12 specimens were prepared for each type of cement paste. At each leaching time (7, 14, 21, and 28 days), three specimens were taken to measure the hydraulic conductivity, and the average of these three measured values was used as the hydraulic conductivity at that time point. For the SEM observation, at least three different locations on the leached specimens were examined, and representative images were captured. XRD and NMR analyses were performed once on the sampled locations. The results of XRD and NMR analyses were highly consistent across repeated measurements.

The phase composition was analyzed using X-ray diffraction (XRD). The X-ray diffractometer used is the Ultima IV model produced by RIGAKU Corporation in Tokyo, Japan. In this study, the samples were scanned over the 2θ range of 5–70. The step size was 0.01°, and the scanning speed was 2°/min.

The pore structure was determined using a nuclear magnetic resonance instrument. This experiment used the MesoMR12-040H-I nuclear magnetic resonance instrument produced by Suzhou Neway Analytical Instrument Co., Ltd. (Suzhou, China). The echo time for this test was 0.20 ms, with 64 scans. The aperture distribution was obtained by inverting the transverse relaxation time T2 spectrum, with a T2 range of 0.01~10,000 ms.

The apparent morphology was observed using a scanning electron microscope. The equipment used is the SU3500 scanning electron microscope produced by Hitachi (Tokyo, Japan). The acceleration voltage used in this test was 5 kV, the working distance was 8 mm, and the magnification was 20,000 times.

**Figure 1:**
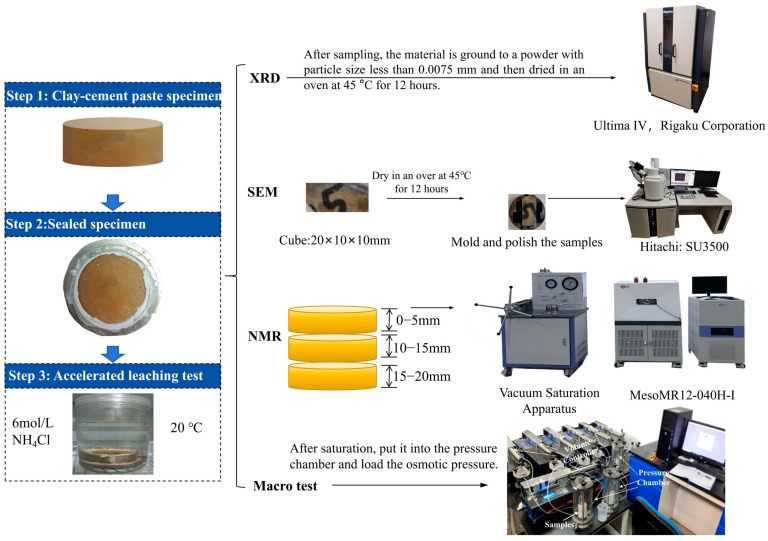
Test flow chart.

The permeability coefficient was measured using a fully automatic permeability tester for cement-based materials. This instrument, manufactured by TKA (Taikeao Company, Aomori, Japan), consists of seven sets of controllers and three pressure chambers, enabling simultaneous measurement of the permeability coefficients of three specimens. Prior to the measurement, the specimens were vacuum-saturated: the vacuuming lasted for 8 h at a pressure of –0.09 MPa. After vacuuming, deionized water was introduced, and the specimens were saturated under atmospheric pressure for one day to eliminate the influence of entrapped air on the test results. During permeability measurement, the confining pressure was set to 1 MPa and the seepage pressure to 200 kPa. The experimental procedure is shown in Figure 1.

Both ammonium nitrate and ammonium chloride solutions could be adopted in the accelerated leaching test of cement-based materials. From a chemical perspective, both NH_4_NO_3_ and NH_4_Cl are weak acid salts that accelerate the leaching of calcium by lowering the pH of the pore solution and disrupting the solid-liquid equilibrium of hydrated products. However, their quantitative differences lie in the solid-liquid equilibrium constants and the critical decalcification concentrations. For instance, as reported by Phung et al. [1], the critical concentration for noticeable solid-phase calcium decomposition in cementitious materials is approximately 273 mol/L, 2320 mol/L, and 2730 mol/L for 6 mol/L NH_4_NO_3_, whereas it is approximately 39 mol/L, 2681 mol/L, and 3000 mol/L for 6 mol/L NH_4_Cl [47]. Regarding the selection of NH_4_Cl in this study instead of NH_4_NO_3_, our primary consideration was experimental feasibility and safety compliance. Previous studies (e.g., Refs. [31,32,33]) have demonstrated its reliability in simulating the leaching process of cementitious materials.

The specimens were cylindrical, with a diameter of 50 mm and a thickness of 20 mm. A total of 12 replicate specimens were prepared, and each specimen was placed independently in a separate leaching bottle containing 1000 mL of 6 mol/L NH_4_Cl solution, which corresponds to 6 mol of NH_4_Cl per bottle. Based on our estimation, the total amount of reactive phases (CH and C-S-H) in a single specimen is far less than 0.1 mol, indicating that the NH_4_Cl supply is substantially excessive throughout the leaching process; hence, no solution replacement was required during the 28-day test period.

The leaching duration was set to 28 days because accelerated leaching tests on cementitious materials are commonly conducted for one month or longer in the literature [2,12], and considering that the clay–cement paste used in this study is inherently less resistant to leaching than plain cement paste, a 28-day period is considered sufficient to capture the leaching behavior effectively.

## 3. Experimental Result Analysis

### 3.1. Content of Cementitious Materials

**Figure 2:**
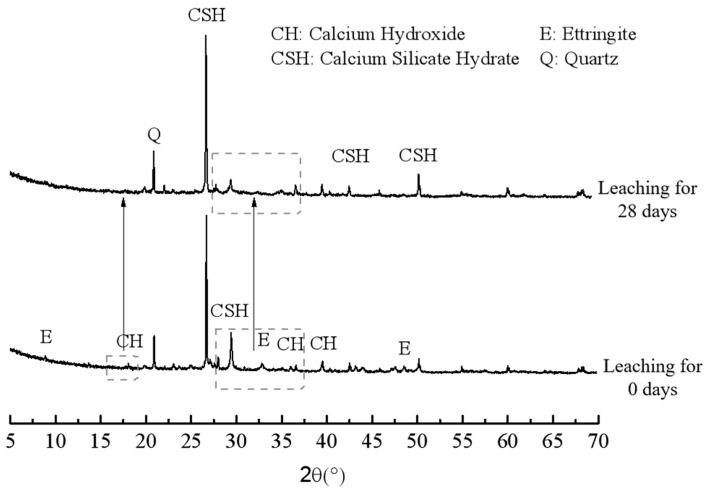
XRD result comparison of intact and leached clay–cement samples.

By comparing the changes in cement hydration products before and after leaching, the leaching process can be elucidated. Figure 2 presents the XRD patterns of intact and leached cement-clay slurry specimens, measured over a diffraction angle (2θ) range of 5° to 70°. The leaching duration was 28 days. As shown in the figure, the intact specimens contain abundant cement hydration products, including calcium hydroxide (CH), calcium silicate hydrate (C-S-H gel), and a small amount of ettringite (AFt). The main diffraction peaks of CH are located at approximately 18°and 34°. A broad hump background is observed between 29°and 35°, indicating the presence of abundant C-S-H gel. The main ettringite peak appears around 9.1°, with additional peaks in the 45–50° range; however, these peaks are less intense than those of C-S-H gel and CH, suggesting relatively low crystallinity. After 28 days of leaching, the main CH and ettringite peaks almost completely disappear, indicating that both hydration products have been leached. The hump peak corresponding to C-S-H gel shows a marked decrease in intensity, replaced by a higher main peak at approximately 24°, which may be attributed to the decalcification of C-S-H gel with a high calcium-to-silicon ratio. In addition, a diffraction peak corresponding to quartz appears in the pattern after leaching, which may also result from the decomposition of C-S-H gel.

### 3.2. NMR Analysis

**Figure 3:**
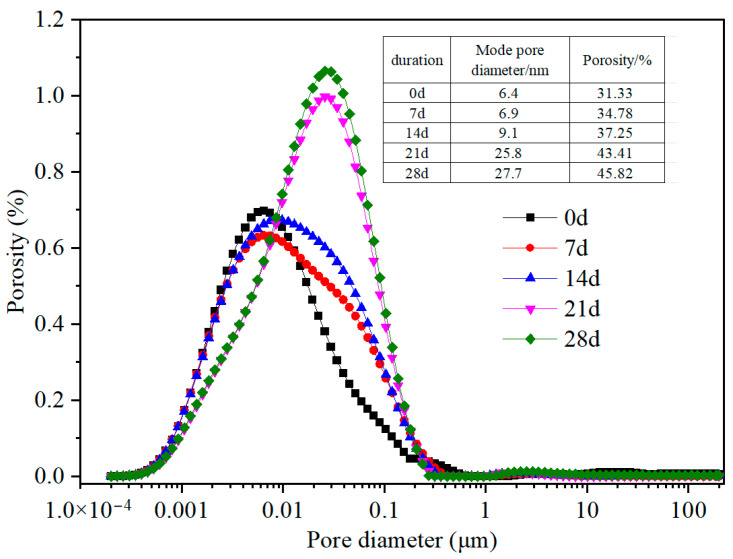
Pore size distribution curve evolution of clay–cement paste.

Figure 3 illustrates the evolution of the pore size distribution of clay–cement paste specimens during the leaching process. As shown in the figure, with increasing leaching duration, the pore size distribution curves shift to the right, indicating pore coarsening and an increase in overall porosity. Between 14 and 21 days of leaching, the porosity of the specimens sharply increases from 0.372 to 0.434, the peak pore diameter increases from 9.1 nm to 25.8 nm, and the corresponding differential volume fraction at the peak pore diameter increases from 0.7% to 1.0%. This accelerated deterioration may be attributed to the leaching-induced penetration of the specimens. Compared with the undissolved specimens, after 28 days of leaching duration, the overall porosity increases from 0.313 to 0.458, and the peak pore diameter increases from 6.4 nm to 27.7 nm.

**Figure 4:**
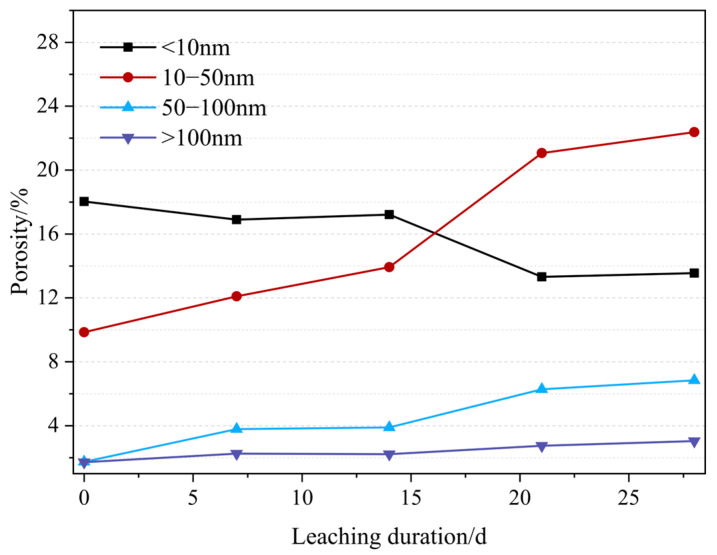
Different pore percentage evolution curves of clay–cement paste.

To better reveal changes in the material’s pore structure, Figure 4 presents the evolution curves for gel pores (<10 nm), small capillary pores (10–50 nm), large capillary pores (50–100 nm), and macropores (>100 nm) during calcium leaching. It can be observed that the proportion of gel pores gradually decreases with leaching duration, from 18% to 14% after 28 days. In contrast, the proportions of small capillary pores, large capillary pores, and macropores gradually increase. Among these, the proportion of small capillary pores shows the largest increase, from 10% to 22.1%. In contrast, the proportions of large capillary pores and macropores increase more modestly, from 1.73% to 3.03% and from 1.71% to 3.03%, respectively. The decrease in the proportion of gel pores is mainly attributed to the decomposition of C-S-H gel. Meanwhile, the increase in small and large capillary pores is due, on the one hand, to the decomposition of CH and, on the other hand, to the enlargement of pores resulting from the decomposition of gel pores, which generates some capillary pores.

**Figure 5:**
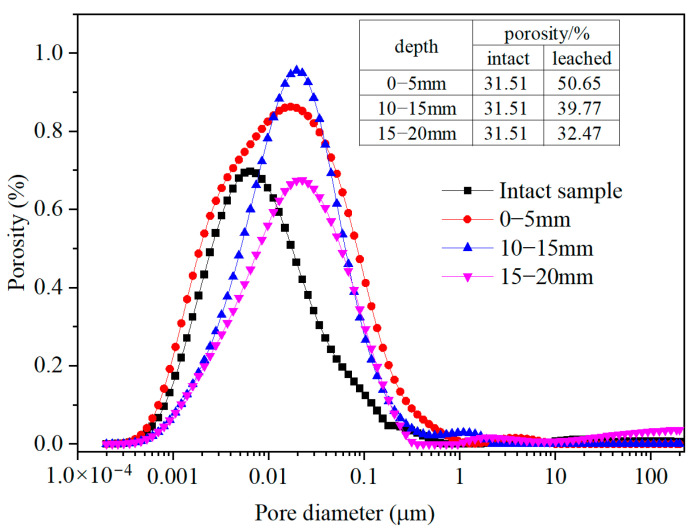
Evolution of pore size distribution curves of clay–cement paste at different positions after 28 days leaching duration.

Figure 5 illustrates the changes in pore structure at different locations of the clay–cement paste after 28 days of calcium leaching. The sampling depths were 0–5 mm, 10–15 mm, and 15–20 mm, designated as the upper, middle, and lower parts, respectively. The pore size distribution curve of the undissolved specimen exhibits a unimodal normal distribution. After calcium leaching, the pore size distribution remains normally distributed; however, the curve becomes significantly broader, the peak height increases, and the peak pore diameter increases. This indicates that calcium leaching increases the specimen’s total porosity, widens the pore-size distribution range, and causes pore coarsening. Notably, for the upper specimen (0–5 mm), a slight increase in pore volume is observed in the range of 1–10 nm. In contrast, such an increase is not evident in the middle and lower specimens, indicating that calcium leaching in these parts is significantly lower than in the upper part. The middle specimen exhibits a peak pore diameter of 10.5 nm with a corresponding proportion of 0.95%, which is higher than that of the upper specimen (10.3 nm, 0.87%). However, in the pore size ranges of <10 nm and >100 nm, the pore proportion of the upper specimen is larger than that of the middle specimen. This suggests that as calcium leaching increases, the quantities of both gel pores and capillary pores increase simultaneously. The increase in capillary pores is primarily attributed to the preferential leaching of portlandite (CH). Under the attack of NH_4_Cl solution, CH crystals are rapidly consumed, leaving cavities that directly translate into an increased capillary pore volume. Conversely, the apparent increase in gel pores does not result from additional C-S-H formation, but from the structural reorganization and decalcification of C-S-H gel. As calcium ions are leached out, the Ca/Si ratio decreases, leading to changes in interlayer spacing and the opening of previously closed micropores within the gel matrix. This results in a measured increase in the gel-pore range. After 28 days of leaching duration, the porosities of the upper, middle, and lower specimens increase from 31.33% to 50.65%, 39.77%, and 32.47%, respectively.

**Figure 6:**
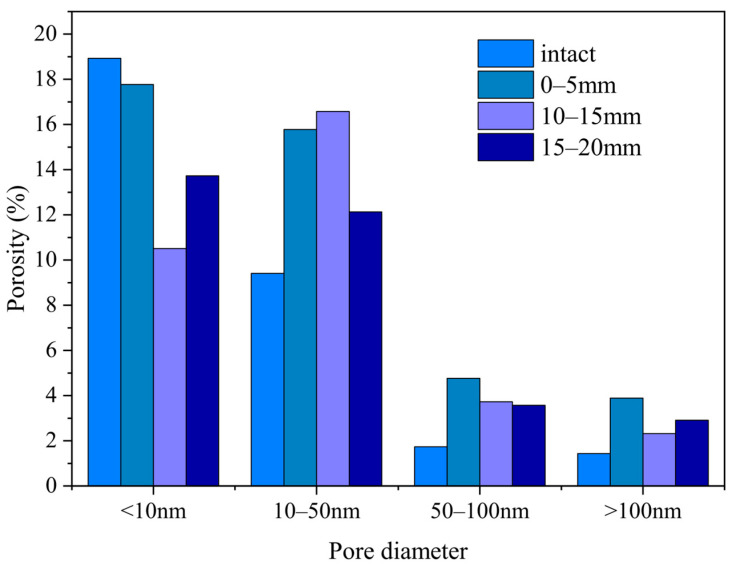
Pore percentage evolution curves of clay–cement paste at different positions after 28 days leaching duration.

Figure 6 presents the pore proportion distribution at different locations after 28 days of leaching duration. As shown in the figure, the undissolved specimen has the highest gel pore proportion at 18.93%, while the middle and lower specimens have lower proportions of 10.51% and 13.73%, respectively. In contrast, the proportions of small capillary pores are higher in the middle and lower specimens, reaching 16.58% and 12.12%, respectively. These differences may be attributed to the fact that, in the early stage of calcium leaching, the decomposition of cementitious materials is dominated by calcium hydroxide (CH), and the pores generated by CH decomposition are mainly capillary pores. This leads to an increase in the proportion of capillary pores and a decrease in the proportion of gel pores in mildly dissolved regions. As leaching progresses, the C-S-H gel in the cementitious materials also begins to decompose, leading to further increases in both gel pores and capillary pores, reaching 17.77% and 15.77%, respectively. For macropores larger than 100 nm in diameter, their proportions remain consistently low across all specimens, generally below 4%.

### 3.3. Surface Morphology

**Figure 7:**
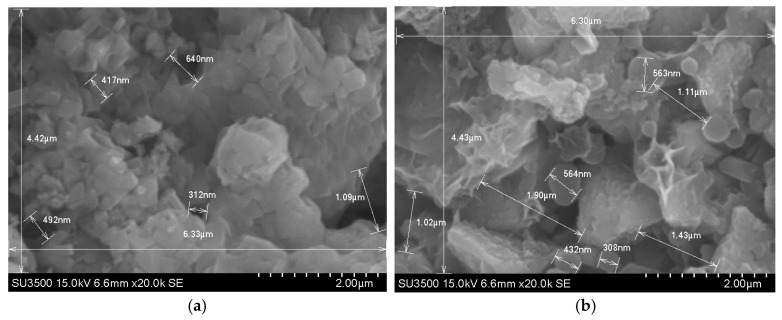
Scanning electron microscope images of clay–cement paste surface: (**a**) intact sample; (**b**) leached sample.

Figure 7 presents the scanning electron microscopy (SEM) images of the surface of intact and 28-day-leached clay–cement paste specimens at a magnification of 20,000×. The SEM images clearly reveal the leaching of cementitious phases (particularly CH), which qualitatively corroborates the increased meso-/macro-pore proportion indicated by the NMR T_2_ distribution shifts. To move beyond purely qualitative observation, we performed semi-quantitative image analysis and measured at least 10 representative pores/particles from each image. For the intact specimen, the measured pore sizes ranged from 312 nm to 1.09 μm (average ~0.59 μm), with a few isolated pores up to 1.09 μm; the surface exhibited well-defined, large CH crystal aggregates and a continuous flocculent C-S-H gel network. After 28 days of leaching, the image analysis revealed a marked increase in both pore population and size: pore diameters ranged from 308 nm to 1.90 μm, with the larger pores reaching 1.90 μm (average ~914 μm), while the CH crystals were severely eroded, leaving only fibrous and spherical remnants, indicating the loss of cementitious properties. These quantified microstructural changes are consistent with the NMR T_2_ distribution shifts, providing robust evidence of leaching-induced degradation.

### 3.4. Hydraulic Conductivity

**Figure 8:**
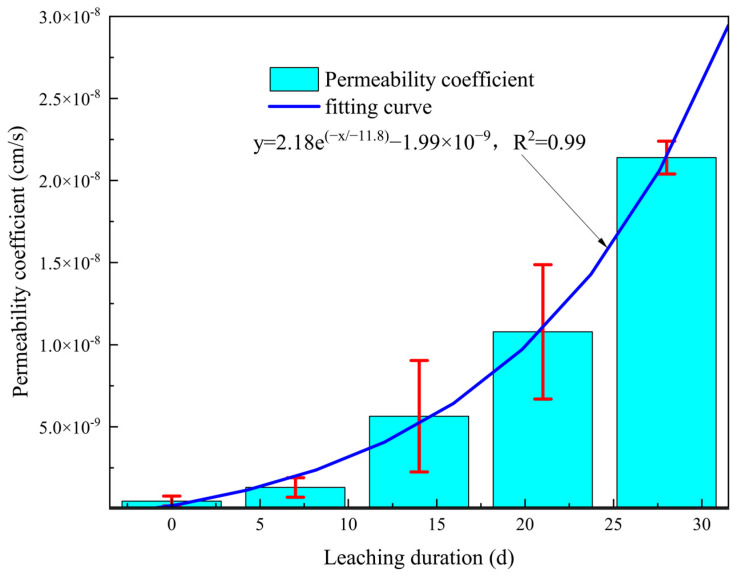
Hydraulic conductivity evolution of clay–cement pastes.

Figure 8 presents the evolution data of hydraulic conductivity during the leaching process, along with the corresponding fitted curve. As shown in the figure, the initial hydraulic conductivity of the clay–cement grout specimen is 4.7 × 10^−10^ cm/s. After 28 days of leaching, the hydraulic conductivity increases to 2.14 × 10^−8^ cm/s, a two-order-of-magnitude increase, indicating that calcium leaching significantly impairs the material’s anti-seepage capacity. Based on the measured data, the hydraulic conductivity of the cement-clay grout is fitted using an exponential equation, yielding the fitted curve y = 2.18 × 10^−9^ × e^−t/11.8^ − 1.99 × 10^−9^. The coefficient of determination (R^2^) is 0.99, suggesting good fitting quality. The trend of the fitted curve indicates that the hydraulic conductivity increases at an accelerating rate as the leaching process progresses. Therefore, during engineering operations, once the anti-seepage performance of the cement grouting material deteriorates due to calcium leaching, timely measures should be taken to prevent engineering safety incidents.

**Figure 9:**
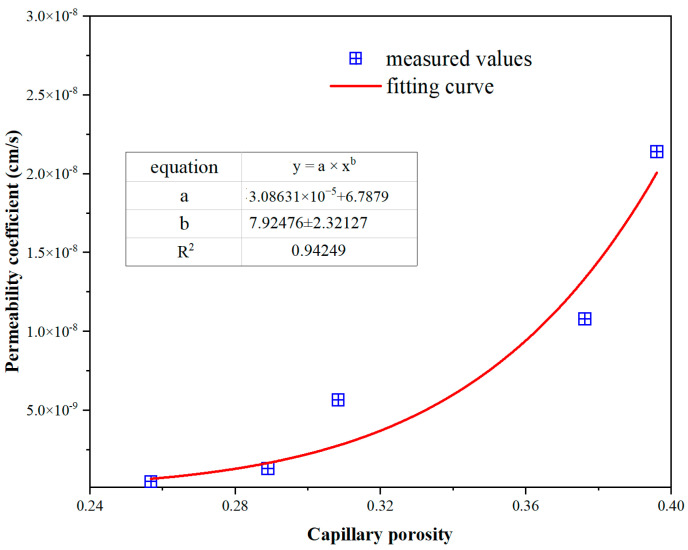
Evolution of permeability coefficient with porosity.

In the simulation of calcium leaching from cement-based materials, the evolution of material porosity can be determined by calculating the amount of dissolved cementitious substances. However, to perform coupled permeability–leaching analysis and solve the advection–diffusion equation and the seepage continuity equation, it is necessary to establish the relationship between porosity and hydraulic conductivity. Hydraulic conductivity is commonly defined as a function of capillary porosity. To this end, based on previously obtained data on material porosity evolution and the hydraulic conductivity measured in this section, the correlation between capillary porosity and hydraulic conductivity is calibrated, as shown in Figure 9. A power-law function is adopted in this study, and the fitted curve is y = 3.08 × 10^−5^ × x^7.92^. The coefficient of determination, R^2^ = 0.94, indicates a satisfactory fitting result.

### 3.5. Porosity-Derived Diffusivity

In diffusion-driven leaching, calcium ion precipitation mainly depends on the concentration difference between the inside and outside, and the precipitation rate is closely related to the diffusion coefficient of cement-based materials. Van Eijk and Brouwers [48] proposed an improved relationship between porosity and the effective diffusion coefficient based on previous work, as shown in Equation (1):

(1) $$\frac{D_{e}}{D_{0}}=0.0025-0.07\varphi_{\mathrm{cap}}^{2}-0.18H\left(\varphi_{\mathrm{cap}}-0.18\right){\left(\varphi_{\mathrm{cap}}-0.18\right)}^{2}+0.14\varphi_{\mathrm{cap}}^{2}+3.6H\left(\varphi_{\mathrm{cap}}-0.16\right){\left(\varphi_{\mathrm{cap}}-0.16\right)}^{2}$$

where $D_{e}$ is the effective diffusion coefficient of the sample (m^2^/s), $D_{0}$ is the diffusion coefficient of calcium ions in water (m^2^/s), $\varphi_{\mathrm{cap}}$ is the capillary porosity, and *H*( ) denotes the weighting function, taking the value 1 for x > 0 and 0 for x < 0.

**Figure 10:**
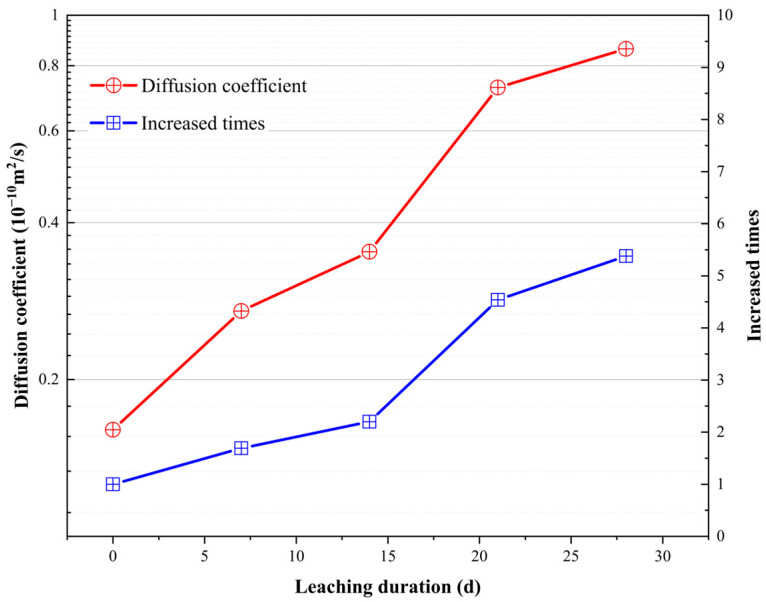
Diffusion coefficient evolution and increase times of clay–cement paste specimens.

The porosity-derived diffusivity was estimated using the semi-empirical relationship proposed by Van Eijk and Brouwers [48], which correlates the transport property with the measured open porosity. This model was chosen owing to its use by numerous researchers in calcium leaching modeling. The results are presented in Figure 10. The figure shows that the material’s initial diffusion coefficient is 1.6 × 10^−11^ m^2^/s, which increases rapidly with leaching time. After 28 days of leaching, the diffusion coefficient eventually reaches 8.6 × 10^−11^ m^2^/s, corresponding to an increase factor of 5.3. As reported in the preceding text, the permeability coefficient of the specimens increased by approximately two orders of magnitude. This result indicates that under leaching conditions dominated by diffusion, the degradation rate of the cement-clay paste is slower than that under conditions governed by the combined action of permeation and diffusion.

**Figure 11:**
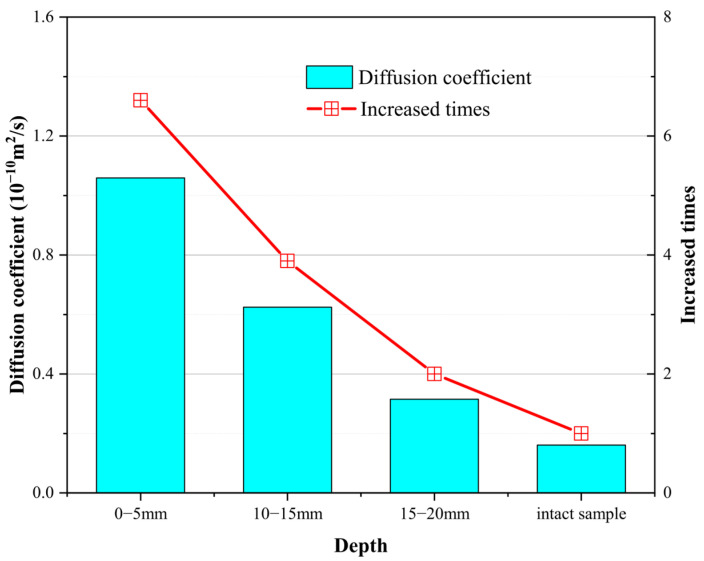
Different increase times of cement–paste specimens.

The diffusion coefficients and their increase factors at different positions of the cement-clay paste are shown in Figure 11. The figure shows that both the diffusion coefficient and its increase factor are closely related to position. The closer to the surface, the larger the diffusion coefficient and the increase factor. After 28 days of leaching, the diffusion coefficient of the specimen in the 0–5 mm range increases to 1.05 × 10^−10^ m^2^/s, while those in the 10–15 mm and 15–20 mm ranges are 6.24 × 10^−11^ m^2^/s and 3.14 × 10^−11^ m^2^/s, respectively. Relative to the initial diffusion coefficient of 1.6 × 10^−11^ m^2^/s, the increase factors from the outer to the inner part are 6.6, 3.9, and 2.0, respectively. The evolution of the diffusion coefficient and its increase factor is consistent with the increase in porosity of the material reported in the preceding text.

## 4. Mechanism Analysis

Cement-clay slurry is an anti-seepage material prepared by mixing cement and clay. During the mixing process, dicalcium silicate and tricalcium silicate in the cement undergo hydration reactions, producing calcium hydroxide and calcium silicate hydrate, which bind the clay particles together. Under prolonged exposure to environmental water, calcium hydroxide is preferentially dissolved and leached out, leading to an increase in the number of capillary pores. As the calcium ion concentration in the pore solution continuously decreases, the calcium silicate hydrate gel (C-S-H gel) gradually begins to decompose, resulting in a sustained reduction in the calcium-to-silicon ratio and the generation of both gel pores and capillary pores. Since the decomposition of calcium hydroxide and calcium silicate hydrate both contribute to the formation of capillary pores, the material’s diffusion and permeability coefficients increase accordingly, thereby accelerating the leaching of calcium ions and promoting the progressive development of the leaching process.

From the above leaching mechanism, it is evident that a substantial increase in capillary pores is a key factor accelerating the leaching process. The sources of capillary pores include the decomposition of calcium hydroxide and that of calcium silicate hydrate. From the perspective of reaction activation energy, the decomposition rate of calcium hydroxide is significantly higher than that of calcium silicate hydrate. In contrast, the leaching rate of the material increases with the degree of leaching. Therefore, reducing the content of capillary pores in the early stage of leaching is particularly critical.

As we can infer from the hydraulic conductivity and diffusion coefficient, the effective diffusion coefficient increased by approximately 5.3-fold (from 1.6 × 10^−11^ m^2^/s to 8.6 × 10^−11^ m^2^/s), while the hydraulic conductivity increased by approximately 45.5-fold (from 4.7 × 10^−10^ cm/s to 2.14 × 10^−8^ cm/s, i.e., nearly two orders of magnitude).

According to the capillary model and Poiseuille’s law, the hydraulic conductivity (K) is proportional to the square of the effective pore radius (R^2^), whereas the effective diffusion coefficient (D) is primarily governed by the tortuosity (τ) and porosity (ϕ) via the relationship D ∝ ϕ/τ, exhibiting a much weaker dependence on a single pore size dimension. During leaching, the dissolution of calcium-bearing phases preferentially occurs at the narrow pore constrictions (ink-bottle necks). Even a modest increase in the throat radius is magnified quadratically in hydraulic conductivity, leading to an orders-of-magnitude jump (45.5-fold in our case).

The diffusion coefficient reflects the average tortuosity of the entire pore network—that is, the mean tortuous path length for ionic migration through both connected and isolated pores. The 5.3-fold increase in D suggests that the overall tortuosity did not undergo a catastrophic reduction, because a substantial fraction of “dead-end” pores still contribute to porosity without effectively shortening the mean transport path.

In contrast, hydraulic conductivity is exceptionally sensitive to the topological connectivity of the pore channels. Once the leaching solution dissolves and opens the critical bottlenecks that connect adjacent pores, the system crosses the percolation threshold, leading to a sudden formation of continuous fluid pathways. This topological transition can trigger a sharp surge in hydraulic conductivity (45.5-fold) without requiring a correspondingly dramatic change in the bulk porosity or average tortuosity. The observed decoupling—5.3-fold increase in D versus 45.5-fold increase in K—is therefore attributed to the combined influence of pore-radius scaling and connectivity percolation.

**Figure 12:**
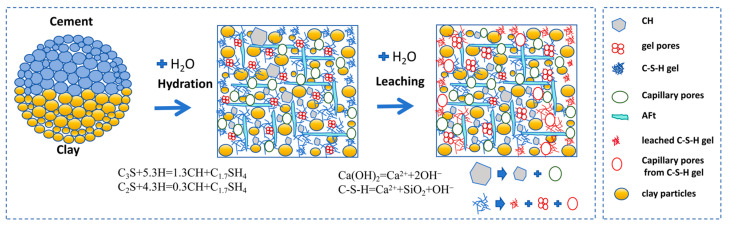
Calcium leaching mechanism of hardened clay–cement paste.

Despite the systematic findings of this study regarding the permeability evolution of clay–cement composites under leaching, several limitations should be acknowledged and warrant further investigation. The NMR pore size analysis relies on the assumption of a constant surface relaxivity (ρ_2_), which may be subject to drift due to leaching-induced mineralogical changes (particularly CH dissolution and C-S-H decalcification). Consequently, the derived pore size distributions should be interpreted as equivalent estimates under this assumption, and future calibration using MIP or BSE-IA is desirable. The Van Eijk and Brouwers porosity model, originally established for cement pastes, was adopted here as a first-order approximation without direct experimental validation for clay–cement composites. Constrained by sample availability and equipment limitations, 3D quantitative pore connectivity characterization using Micro-CT or MIP was not performed, leaving the topological evolution of the pore network insufficiently understood. This study was limited to a single clay type, a fixed mix proportion, and a relatively short leaching period (28 days), so caution should be exercised when generalizing the conclusions to other clay sources or long-term service conditions. These limitations have been explicitly stated and prioritized as key directions for our future research (Figure 12).

## 5. Conclusions

In this paper, accelerated calcium leaching tests were conducted on clay–cement pastes. A variety of techniques, including XRD, SEM and NMR, were used to characterize the microstructural and mineralogical changes of leached samples. The effect of accelerated leaching on transport behavior was studied by measuring changes in water permeability and calculating diffusivity. The main conclusions are as follows:

(1) The leaching process degrades the clay–cement paste, primarily manifesting as the decomposition of cementitious materials. XRD analysis shows that after 28 days of leaching, the characteristic peaks of portlandite and ettringite almost completely disappear, while the peak intensity of C-S-H gel significantly decreases and shifts, indicating decalcification and decomposition. SEM images further confirm that the portlandite crystals on the sample surface are severely damaged after leaching, the C-S-H gel loses its cementitious properties, and the number of pores increases with a notable increase in pore size (reaching a maximum of 1.90 μm).
(2) NMR results indicate that with increasing leaching time, both the total porosity and peak pore size of the clay–cement paste increase significantly, suggesting intensified pore coarsening. The proportion of gel pores (<10 nm) gradually decreases. In comparison, the proportion of small capillary pores (10–50 nm) increases substantially (from 10% to 22.1%), mainly due to the decomposition of C-S-H gel and CH. After 28 days of leaching, the degree of degradation varies distinctly along the sample: the total porosity of the upper layer (0–5 mm) rises from 31.33% to 50.65%, with both gel and capillary pores increasing; in contrast, the middle and lower layers exhibit significantly lower calcium leaching and limited porosity growth. These evolutionary characteristics of the pore structure reveal the progressive deterioration pattern induced by leaching on the material’s microstructure.
(3) Under the effect of leaching, the hydraulic conductivity of the specimens increases at an accelerating rate with the leaching process, rising from an initial value of 4.7 × 10^−10^ cm/s to 2.14 × 10^−8^ cm/s after 28 days, an increase of two orders of magnitude. Meanwhile, the diffusion coefficient of the material increases from 1.6 × 10^−11^ m^2^/s to 8.6 × 10^−11^ m^2^/s, corresponding to a 5.3-fold increase, indicating a slower degradation rate than that of hydraulic conductivity. Both the diffusion coefficient and its increase factor gradually decrease from the surface to the interior of the specimen, consistent with the evolution pattern of porosity.

Based on the experimental findings of this study, we offer the following engineering recommendations for the application of clay–cement composites as anti-seepage materials in dam constructions: (1) During the design phase, particular attention should be paid to the core wall and the bottom of curtain grouting zones, where the combined action of high hydraulic head and long-term leaching is most pronounced. (2) During operation and maintenance, given that the hydraulic conductivity of the clay–cement composite increases at a much faster rate than the diffusion coefficient under leaching (45.5-fold increase in K versus 5.3-fold in D in this study), it is advisable to prioritize the real-time monitoring of seepage pressure and leakage rate at these vulnerable locations to provide early warning against leaching-induced seepage failure, particularly piping and soil flow phenomena.

## Figures and Tables

**Table 1:** Chemical composition of the cement (%) [46].

Compound	Content	Compound	Content
CaO	67.58	SO_3_	2.63
SiO_2_	13.57	Cl^−^	0.021
Al_2_O_3_	5.05	Na_2_O	0.04
Fe_2_O_3_	3.64	Loss on ignition	0.37
MgO	1.79	Insoluble residue	2.39

**Table 2:** Cement quality from the manufacture fact sheet.

Cement Type	Specific Surface Area (m^2^/kg)	Plaster	Mixed Material	Setting Time (h:min)		Compressive Strength (MPa)		Flexural Strength (MPa)	
				Initial Setting	Final Setting	3 d	28 d	3 d	28 d
P.O42.5	359	5%	16%	194	245	28.3	51	6.1	8.7

**Table 3:** Chemical composition of the clay (%).

Compound	Content	Compound	Content	Compound	Content
SiO_2_	59.90	MgO	1.35	BaO	0.10
Al_2_O_3_	19.10	TiO_2_	1.18	P_2_O_5_	0.08
Fe_2_O_3_	9.04	CaO	0.84	SO_3_	0.03
K_2_O	4.20	Na_2_O	0.71	Cr_2_O_3_	0.03
CO_2_	3.16	MnO	0.16	ZnO	0.02

**Table 4:** Mix proportion of the slurry.

Slurry Type	Cement/kg	Clay/kg	Water/kg	Water Cement Ratio	Water Powder * Ratio
Clay–cement slurry	50.00	50.00	50.00	1.00	0.50

* “Powder” denotes the combined mass of (Cement + Clay).

## Data Availability

The original contributions presented in this study are included in the article. Further inquiries can be directed to the corresponding authors.

## Abbreviations

The following abbreviations are used in this manuscript:

CH	Calcium Hydroxide
C-S-H	Calcium Silicate Hydrate gel
AFt	Ettringite
SEM	Scanning Electron Microscopy
NMR	Nuclear Magnetic Resonance
XRD	X-ray diffraction
C_2_S	2CaO·SiO_2_
C_3_S	3CaO·SiO_2_
